# Interobserver reproducibility and interocular symmetry of the macular ganglion cell complex: assessment in healthy children using optical coherence tomography

**DOI:** 10.1186/s12886-020-01379-z

**Published:** 2020-05-24

**Authors:** Alicia Muñoz-Gallego, Javier De la Cruz, Martín Rodríguez-Salgado, José L. Torres-Peña, Javier Sambricio, Ana Ortueta-Olartecoechea, Pilar Tejada-Palacios

**Affiliations:** 1 Ophthalmology Department, Hospital Universitario 12 de Octubre, Madrid, Spain; 2 Servicio Madrileño de Salud (SERMAS), Primary Health Care, Madrid, Spain; 3 Hospital Universitario 12 de Octubre, Healthcare Research Institute (IMAS12), Servicio Madrileño de Salud (SERMAS-H12O), CAA-6D. Avda. de Códoba s/n, E-28041 Madrid, Spain; 4 Spanish Network for Research in Mother&Child Health and Development (RED SAMID RD16/0022/0011), Instituto de Salud Carlos III (ISCIII), Madrid, Spain; 5 Complutense University of Madrid (UCM), Madrid, Spain

**Keywords:** Optical coherence tomography, Ganglion cell complex, Retinal ganglion cells, Macular ganglion cell-inner plexiform layer, Macular retinal nerve fibre layer, Childhood, Paediatric reference database, Reproducibility, Interobserver reproducibility, Symmetry, Interocular symmetry

## Abstract

**Background:**

Assessment of interobserver reproducibility and interocular symmetry using optical coherence tomography (OCT)–based measurements of the macular ganglion cell complex (GCC) in healthy children facilitates interpretation of OCT data. We assessed the interobserver reproducibility and interocular symmetry of GCC and evaluated candidate determinants.

**Methods:**

This was a cross-sectional study performed in a primary and tertiary health-care setting. A total of 126 healthy participants aged 5 to 18 years were eligible. GCC scans were performed by 4 operators using the Topcon 3D OCT-2000 device. Intraclass correlation coefficients (ICCs) were used to estimate reproducibility and symmetry. Cut-off points for symmetry were defined as the 95th percentile of the absolute interocular difference for 6 GCC parameters. Percentile distributions of interocular difference were generated based on age and difference in absolute interocular spherical equivalent (SE).

**Results:**

The reproducibility ICC ranged from 0.96 to 0.98 for all 6 GCC parameters. Cut-off points for interocular symmetry of the superior and inferior quadrants and total macular retinal nerve fibre layer thickness (mRNFL) and macular ganglion cell layer-inner plexiform layer thickness were 3.5, 4.5, 3.0, 3.0, 2.5, and 2.5 μm respectively. A positive association was observed between the absolute interocular difference of SE and superior and total mRNFL symmetry values (*p* = 0.047 and *p* = 0.040, respectively).

**Conclusions:**

OCT measurements of GCC in healthy children show excellent reproducibility. Interocular differences in SE should be assessed when mRNFL differences exceed the 95% cut-off. These findings can contribute to establish reference values for interocular symmetry in paediatric GCC parameters.

## Background

Optical coherence tomography (OCT) is a rapid, noninvasive, noncontact method that plays an increasingly important role in paediatric ophthalmology [1]. It uses low-coherence interferometry to perform high-resolution cross-sectional imaging of tissue morphology, thus providing an optical biopsy [2]. The introduction of the spectral domain (SD-OCT) in 2006 improved both precision and resolution [1]. Topcon 3D OCT-2000 (Topcon Corporation, Tokyo, Japan) is an SD-OCT device that includes a fully automated OCT layer segmentation algorithm, TABSTM (Topcon Advanced Boundary SegmentationTM), which is now included in its OCT FastMapTM software.

All OCT devices have an integrated normative database, which includes data only from individuals aged ≥18 years [3]. The OCT has become an essential tool in scientific and clinical practice in ophthalmology. In particular, the macular ganglion cell complex (GCC), which comprises macular ganglion cell layer-inner plexiform layer thickness (GCL-IPL) and macular retinal nerve fibre layer thickness (mRNFL), is a potential marker for glaucoma, optic nerve disease, and neurological diseases and progression [4, 5]. To evaluate changes in retinal and optic nerve measurements accurately, it is first necessary to determine the range in the normal population and to quantify the accuracy, reproducibility, and repeatability of measurements made by the system [6]. Reliability and repeatability of diagnostic tools is very important, especially in children, because collaboration is limited. Numerous authors to date have investigated the reproducibility of OCT in macular and optic nerve measurements in healthy children [3, 7–13]. To our knowledge, few studies have researched the values of GCL-IPL in healthy children, with one in children with refractive errors [14], one in healthy Turkish children [15], one in healthy Spanish children [16], and another that compared the result obtained in healthy children with the results for children with congenital glaucoma [17]. However, none of these studies included reproducibility analyses. Only 1 article included reproducibility of GCL-IPL measurements with a handheld OCT device in children with optic pathway gliomas [18].

The objectivity and reproducibility of Topcon OCT have been proven by several authors in healthy adults, but never in children [19–23].

Interocular asymmetry of retinal biometric parameters may be indicative of disease [24]. However, it is important to know the extent to which these parameters can be asymmetric in normal eyes [25]. Although the symmetry of the optic disk and retina in children has been studied [1, 13, 24, 26–29], to the best of our knowledge, no such data are available for GCL-IPL and mRNFL in healthy children. In this report, we used Topcon 3D OCT-2000 (Topcon Corporation, Tokyo, Japan) to examine interocular differences in macular GCL-IPL and RNFL in healthy children aged between 5 and 18 years and to determine which factors affect interocular symmetry. The detection of interocular asymmetry in optic nerve or macula parameters may have diagnostic advantages over using isolated measures from a single eye [30]. An absolute interocular difference in GCL-IPL exceeding normal limits may be indicative of glaucoma [31].

In the current study, we examined the interobserver reproducibility of repeated measurements of GCC and interocular symmetry and their determinants using SD-OCT in healthy children.

## Methods

We performed a cross-sectional observational study of Spanish children aged 5–18 years. The study was approved by the Ethics Committee and Research Board at Madrid University Hospital 12 Octubre, Spain (reference number 13/399). All of the children were enrolled through a primary health care centre and a tertiary referral hospital of the Madrid Regional Health Service (SERMAS) (respectively, *Centro de Salud Almendrales* and *Hospital Universitario 12 de Octubre*, both in Madrid, Spain). All parents and guardians provided their written informed consent. Children aged ≥12 years also provided written informed assent. The study protocols adhered to the guidelines of the Declaration of Helsinki for research involving human participants [16].

Patients underwent a complete ophthalmic examination, including visual-acuity measurement, refractive error measurement with and without cycloplegia, anterior segment examination with slit-lamp biomicroscopy, and dilated fundus and stereoscopic optic disc examination. Images were acquired using fundus photography (Topcon 3D OCT-2000, Topcon Corporation, Tokyo, Japan) under cycloplegic mydriasis.

The inclusion criteria have been previously detailed [16].

The macula 3D scan (V) protocol was used to evaluate the GCC. mRNFL and GCL-IPL thickness were recorded as superior, inferior, and total average components within the 7 mm^2^ area centred on the fovea, with a scan density of 512 (vertical) × 128 (horizontal) scans. For all participants, 2 scans were performed for each eye, and the mean of both measurements was used in the statistical analyses. Only children with good quality scans were included in the study. Good quality scan has been previously defined [16]. A total of 4 observers participated in this study. Two OCT scans were performed on each eye of each patient by 2 different observers. Observer A scanned 228 eyes (45.6%), observer B 103 eyes (20.6%), observer C 71 eyes (14.2%), and observer D 96 eyes (19.2%). The average of the 2 observers’ measurements was computed for each macular scan. Analyses for reproducibility were performed only on the right eye. The average of the 2 macular scans of both eyes was used in the statistical analyses of symmetry. Average symmetry was evaluated using relative and absolute interocular differences of the 6 GCC parameters, including their respective percentiles. Cut-off points for interocular symmetry were defined as the 95th percentile of the absolute interocular differences of the 6 GCC parameters. Symmetry was evaluated by age group and SE based on absolute interocular differences.

The software included in Topcon 3D OCT-2000 compares each measurement with the percentile distribution of an embedded adult database and assigns a colour label for different percentile categories [16]. In order to document the qualitative agreement for paediatric OCT of GCC, we estimated the agreement between both OCT scans categorized according to percentile 5. The proportion of observed agreement (OA) refers to the overall agreement, whereas the specific agreement (SA_p ≤ 5_) quantifies the agreement for measurements ≤5 [32, 33].

### Data analysis

The intraclass correlation coefficient (ICC) was determined to evaluate interobserver reproducibility [25]. The ICC was also determined to estimate interobserver reproducibility for GCL-IPL and mRNFL as a function of age and SE. Qualitative agreement was also evaluated in order to determine whether OCT interobserver reproducibility was also high when the specific colour label was assigned to each measurement.

Symmetry was analysed by calculating mean differences for all parameters, first by subtracting left eye parameters from right eye parameters (with positive differences when right eyes had higher values and negative differences when left eyes had higher values), and second based on the absolute value of the interocular difference (only positive values). The ICC was computed to measure interocular agreement/correlation. Univariate and multivariate linear regression analyses were performed to estimate the interocular differences of GCL-IPL and mRNFL as a function of age and SE, for which the regression coefficient was reported along with the *p* value. A p value < 0.05 indicated statistical significance. The normal ranges for absolute interocular differences were established as the 95th percentile for the GCL-IPL and mRNFL.

Data were analysed using SAS 9.3 (SAS Institute Inc., Cary, NC, USA).

## Results

For the initial assessment, 280 eyes from 140 healthy children aged 5 to 18 years old were consecutively enrolled in the present study. Of the 140 children, 14 (10%) were excluded because they did not fulfil the inclusion criteria [16], leaving a sample of 126 right eyes and 124 left eyes in 126 healthy children with a mean age of 10.26 years (SD, 3.37; range, 5.03 to 17.37). Most children were of European origin (82.5%), and 48.4% (*n* = 61) were male. Mean SE was 0.88 (1.49; range, − 3.38 to 4.63) for right eyes and 0.99 (1.63; range, − 3.63 to 5) for left eyes; mean cylinder was − 0.40 (0.48; range, − 3.00 to 0) for right eyes and − 0.39 (0.51; range, − 3.00 to 0) for left eyes. All results presented hereafter refer to the participant’s right eye (*n* = 126) for reproducibility analyses and both eyes for symmetry analyses (*n* = 124, as 2 participants had a cylinder below − 3.00 in the left eye).

**Fig. 1:**
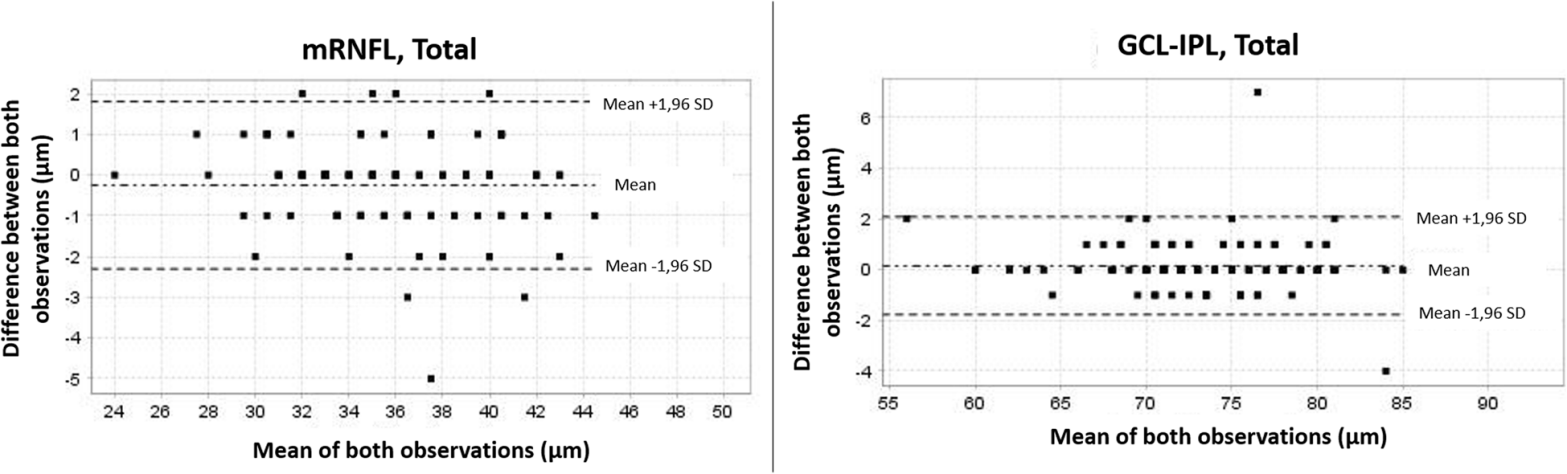
Bland-Altman plot showing the level of interobserver agreement between the optical coherence tomography measurements of the mRNFL and GCL-IPL in children

All GCL-IPL and mRNFL measurements were highly reproducible. ICCs showed almost perfect reliability in all the parameters evaluated (ICCs > 0.95) (Table 1). Qualitative agreement was also evaluated for all measurements, showing a very high OA (ICCs > 0.95). Specific agreement for the detection of measurements less than or equal to the fifth percentile was also high. Figure 1 shows Bland-Altmann plots for the reproducibility of total mRNFL and GCL-IPL between the interobserver measurements. Quantitative reproducibility was also high when analyzed separately by age and SE group, with ICCs above 0.91 in all cases.

**Table 1:** Interobserver reproducibility and agreement of macular fibre complex OCT measurements in healthy children (*n* = 126)

	Quantitative reproducibility		Qualitative agreement		
	ICC (95% CI)	Mean difference (95% CI) (μm)	Observed agreement Percentage (95% CI)	Observations ≤ p5 (n)	Specific agreement ≤ p5 Percentage (95% CI)
**mRNFL**^a^**, Superior**	0.96 (0.94 to 0.97)	−0.14 (− 0.33 to 0.05)	0.98 (0.93 to 1.0)	11	0.84 (0.81 to 0.88)
**mRNFL, Inferior**	0.97 (0.95 to 0.98)	−0,25 (− 0.45 to 0.06)	0.96 (0.91 to 0.99)	11	0.82 (0.78 to 0.86)
**mRNFL, Total**	0.96 (0.94 to 0.97)	−0.25 (− 0.43 to 0.06)	0.99 (0.96 to 1.0)	12	0.96 (0.93 to 0.98)
**GCL-IPL**^b^**, Superior**	0.98 (0.97 to 0.98)	0.17 (−0.03 to 0.37)	0.99 (0.97 to 1.0)	2	0.67 (0.62 to 0.71)
**GCL-IPL, Inferior**	0.97 (0.96 to 0.98)	0.18 (−0.02 to 0.38)	1.0 (0.97 to 1.0)	0	–
**GCL-IPL, Total**	0.98 (0.97 to 0.99)	0.16 (−0.01 to 0.33)	0.99 (0.96 to 1.0)	1	1.00

*ICC* Intraclass correlation coefficient, *SD* Standard deviation, *CI* Confidence interval.

Note. ^a^*mRNFL* Macular retinal nerve fibre layer thickness, ^b^GCL-IPL: macular ganglion cell layer-inner plexiform layer thickness

Global symmetry analyses are shown in Table 2. Interocular symmetry measurements showed high ICCs for all measurements (ICCs > 0.82). ICCs were also high when symmetry was evaluated by age and SE groups (ICCs above 0.92 and 0.91 respectively). Interocular symmetry by age and interocular differences in SE groups are presented in Tables 3 and 4.

**Table 2:** Interocular symmetry of macular fibre complex OCT measurements in healthy children (*n* = 124)

	Right eye Mean (SD) (μm)	Left eye Mean (SD) (μm)	Interocular difference (right eye – left eye), (μm)									Absolute interocular difference, (μm)		ICC (95% CI)
			Mean (95% CI)	*p* value	p2.5	p5	p25	p50	p75	p95	p97.5	p90	p95	
mRNFL^a^, Superior	34.56 (3.78)	34.31 (3.49)	0.26 (− 0.12 to 0.64)	0.182	− 3.5	−2.5	−1.0	0.5	1.5	3.5	4.0	3.0	3.5	0.83 (0.76 to 0.87)
mRNFL, Inferior	37.11 (4.22)	37.61 (4.48)	−0.50 (− 0.88 to 0.13)	0.008	−5.0	−4.0	−1.5	− 0.25	0.5	3.0	3.5	3.5	4.5	0.88 (0.83 to 0.91)
mRNFL, Total	35.83 (3.78)	36.00 (3.80)	−0.17 (− 0.47 to 0.14)	0.282	−3.0	−2.5	−1.0	0	0.5	2.0	3.0	2.5	3.0	0.90 (0.86 to 0.93)
GCL-IPL^b^, Superior	73.56 (5.29)	74.17 (4.74)	−0.60 (−1.06 to 0.15)	0.010	−3.0	−3.0	−1.5	−0.5	0.5	1.5	2.0	2.5	3.0	0.86 (0.81 to 0.90)
GCL-IPL, Inferior	73.22 (4.73)	73.17 (4.64)	0.05 (−0.21 to 0.31)	0.693	−2.5	−2.0	−0.5	0	1.0	2.0	3.0	2.0	2.5	0.95 (0.93 to 0.97)
GCL-IPL, Total	73.38 (4.90)	73.65 (4.63)	−0.09 (−0.03 to 0.20)	0.111	−2.5	−2.0	−1.0	0	0.5	1.5	2.0	2.0	2.5	0,99 (0.99 to 0.99)

*ICC* Intraclass correlation coefficient, *CI* Confidence interval, *SD* Standard deviation, *p* Percentile.

Note. ^a^*mRNFL* Macular retinal nerve fibre layer thickness, ^b^*GCL-IPL* Macular ganglion cell layer-inner plexiform layer thickness

**Table 3:** Interocular symmetry of OCT measurements of GCL-IPL and RNFL in healthy children (age groups)

	*p* value	Age groups (years, y)											
		5–7 y (*n* = 36)			8–10 y (*n* = 38)			11–13 y (*n* = 31)			14–17 y (*n* = 19)		
		Absolute interocular difference (μm)			Absolute interocular difference (μm)			Absolute interocular difference (μm)			Absolute interocular difference (μm)		
		Mean (SD)	p90	p95	Mean (SD)	p90	p95	Mean (SD)	p90	p95	Mean (SD)	p90	p95
**mRNFL**^a^**, Superior**	0.459	1.14 (1.06)	2.50	3.00	1.57 (1.30)	3.50	5.00	1.48 (2.40)	2.00	4.00	1.82 (1.24)	3.50	4.00
**mRNFL, Inferior**	0.517	1.69 (1.56)	4.50	5.00	1.24 (1.21)	3.00	3.50	1.52 (1.88)	3.00	5.00	1.76 (1.29)	3.50	4.00
**mRNFL, Total**	0.943	1.06 (1.03)	2.50	3.50	1.09 (0.99)	3.00	3.00	1.13 (2.14)	2.00	5.00	0.89 (0.72)	2.00	2.50
**GCL-IPL**^b^**, Superior**	0.780	1.08 (0.79)	2.00	3.50	1.08 (0.92)	2.50	3.50	1.60 (4.44)	2.00	4.50	1.34 (0.96)	3.00	3.00
**GCL-IPL, Inferior**	0.543	1.00 (0.89)	2.00	3.50	0.83 (0.77)	2.00	2.50	1.21 (1.67)	2.00	4.00	0.92 (0.75)	2.00	3.00
**GCL-IPL, Total**	0.3291	0.86 (0.77)	1.50	2.50	0.68 (0.70)	1.50	2.00	1.39 (2.95)	2.50	4.50	0.89 (0.72)	2.00	2.00

*SD* Standard deviation, *p* Percentile.

Note: a*mRNFL* Macular retinal nerve fibre layer thickness, ^b^*GCL-IPL* Macular ganglion cell layer-inner plexiform layer thickness

**Table 4:** Interocular symmetry of OCT measurements of GCL-IPL and mRNFL in healthy children (groups of spherical equivalent)

	Absolute interocular difference (μm)									
	Absolute interocular difference of spherical equivalent									
	Whole sample (*n* = 124) SE as continuous variable			Group < 0.75 D (*n* = 110)			Group ≥ 0.75 D (*n* = 14)			*p* value (Groups of SE)
	Intercept (Standard Error)	Regression coefficient (Standard Error)	*p* value	Mean (SD)	p90	p95	Mean (SD)	p90	p95	
**mRNFL**^a^**, Superior**	1.248 (0.177)	0.655 (0.331)	**0.050**	1.36 (1.54)	3.00	3.50	2.25 (1.72)	5.00	5.50	**0.047**
**mRNFL, Inferior**	1.462 (0.171)	0.180 (0.321)	0.577	1.49 (1.48)	3.50	4.50	1.79 (1.76)	4.00	5.00	0.488
**mRNFL, Total**	0.889 (0.150)	0.530 (0.281)	0.061	0.97 (1.29)	2.00	2.50	1.75 (1.58)	4.00	5.00	**0.040**
**GCL-IPL**^b^**, Superior**	1.221 (0.264)	0.087 (0.495)	0.861	1.23 (2.44)	2.00	3.00	1.39 (1.15)	3.50	3.50	0.679
**GCL-IPL, Inferior**	1.053 (0.123)	−0.202 (0.231)	0.383	0.98 (1.11)	2.00	2.50	1.04 (0.97)	2.50	3.50	0.863
**GCL-IPL, Total**	0.935 (0.182)	0.026 (0.342)	0.941	0.92 (1.68)	1.50	2.00	1.14 (0.91)	2.50	3.50	0.446

*D* Diopters, *SD* Standard deviation.

Note: ^a^*mRNFL* Macular retinal nerve fibre layer thickness, ^b^*GCL-IPL* Macular ganglion cell layer-inner plexiform layer thickness

We quantified the impact of age and SE in GCC symmetry. Multivariate regression analysis revealed no statistically significant associations between age and differences in interocular mRNFL and GCL-IPL (*p* > 0.33). A statistically significant correlation was found between the absolute interocular difference in SE groups (SE < 0.75 D and ≥ 0.75 D) and the absolute interocular difference in superior mRNFL (*p* = 0.047) and total mRNFL measurements (*p* = 0.040).

## Discussion

To our knowledge, this study provides the first report on interobserver reproducibility and interocular symmetry of macular GCL-IPL and mRNFL thicknesses, as measured by OCT in healthy children aged 5–18 years. OCT measurements showed excellent interobserver reproducibility. Cut-offs for interocular differences were provided for all 6 GCC parameters. A positive association was observed between the absolute interocular difference of SE and superior and total mRNFL symmetry values.

The main strength of our study was its ability to demonstrate that the Topcon 3D OCT-2000 (Topcon Corporation, Tokyo, Japan) device obtains reproducible measurements in healthy children. The study also investigated the interocular symmetry of GCC measurements in healthy children and established normal limits of symmetry, which ophthalmologists can use in their daily practice. We also demonstrate the absence of statistical associations between age and differences in interocular mRNFL and GCL-IPL. However, a statistically significant correlation was found between the absolute interocular difference in SE and the absolute interocular difference in superior and total mRNFL (*p* = 0.047 and *p* = 0.040, respectively), meaning that in cases of asymmetry, biometric parameters should be assessed in order to determine their influence.

The results of reproducibility and symmetry provided in this study are only applicable to the SD-OCT device Topcon 3D OCT-2000. Measurements are not interchangeable between OCT devices [23, 34–36]. Therefore, direct comparison of GCL-IPL and mRNFL values between various OCT devices may be difficult because of differences in technical specifications, imaging protocols, and thickness measurement algorithms.

Our findings suggest that Topcon OCT shows good interobserver reproducibility when measuring GCC thickness in healthy children. ICCs were higher than 0.9 for all parameters, indicating that this approach is highly reproducible. Our values of interobserver reproducibility are consistent with those of other studies in healthy children based on different OCT devices. Prakalapakorn et al. [9] assessed the reproducibility for pRNFL and macular measurements provided by Stratus OCT (Carl Zeiss, Dublin, California, USA) in 27 healthy and 37 glaucomatous eyes in children and found ICCs higher than 0.9 in macular measurements. Altemir et al. [3] investigated the interobserver and intraobserver reproducibility for macular and optic disc measurements in 100 eyes from 100 healthy children using Cirrus OCT (Humphrey Zeiss Instruments, Dublin, California, USA) and recorded ICCs higher than 0.82 in all measurements. Kiziloglu et al. [37] assessed reproducibility for optic head parameters with iVue 100 SD-OCT (version 3.1; Optovue Inc., Fremont, California, USA) in healthy Turkish children and recorded ICCs higher than 0.9 in all measurements.

We found the worst interobserver reproducibility in the superior quadrant for mRNFL (ICC = 0.96) and inferior quadrant for GCL-IPL (ICC = 0.97), although all ICCs were higher than 0.9.

In contrast with other studies on reproducibility, we analysed this parameter by comparing different groups of age and SE and again found that all ICCs were above 0.9. Analysis of qualitative reproducibility also showed ICCs to be above 0.95, meaning that OCT assigns the same colour label when the measurements are made by 2 different observers. Therefore, Topcon 3D OCT-2000 (Topcon Corporation, Tokyo, Japan) generated reproducible measurements of GCL-IPL and mRNFL in healthy children irrespective of age and SE.

Several studies have used different OCT devices to assess the asymmetry of pRNFL and macular thickness in children and young adults [1, 13, 24, 25, 27, 29, 38]. However, to our knowledge, no previous studies have assessed paediatric interocular asymmetry in macular GCC. We found the mean differences in average mRNFL and GCLT to be < 0.2 μm, whereas the mean differences in the quadrants ranged from 0.26 to 0.50 μm in mRNFL and from 0.05 to 0.60 μm in GCL-IPL. High interocular correlations (> 0.8) were found in all macular parameters. GCL-IPL measurements were more symmetric than mRNFL measurements. Overall, interocular mean differences in macular GCL-IPL and mRNFL measurements were minimal, although large degrees of asymmetry were detected in specific cases. Altemir et al. [25] studied pRNFL and macular symmetry in healthy children by setting the interocular difference tolerance limits for the 2.5th and 97.5th percentiles. These percentiles were used as a reference for the study of pRNFL and macular thickness by Dalgliesh et al.^43^ and by Zhou et al.^5^ for their research on GCC symmetry, both in young adults [5, 38]. Budenz [39] used the 5th and 95th percentiles as tolerance limits of interocular difference to assess pRNFL symmetry in adults. Al-Haddad et al. [1] used the 5th and 95th percentiles to assess macular and pRNFL symmetry in healthy children based on the Cirrus device. As these authors conclude, changes in OCT measurements compared with previous examinations or interocular asymmetry exceeding normal limits should be considered warning signs and an indication for further assessment [25]. All of the abovementioned authors used the relative percentile values and obtained symmetry values by subtracting the value of the left eye from that of the right one. We used the same measurement, but we also determined the absolute symmetry value (always a positive number), as it is easier to interpret. Taking these data as our reference, the cut-off points we established for the superior and inferior quadrants and total mRNFL and GCL-IPL were 3.5 μm, 4.5 μm, 3.0 μm, 3.0 μm, 2.5 μm, and 2.5 μm, respectively, based on the 95th percentile.

Interocular asymmetry of retinal biometric parameters should be evaluated in context with other clinical measures, because large degrees of asymmetry may be found in specific cases [25]. In order to determine whether higher interocular differences could be explained by higher interocular differences in SE or age groups, symmetry was evaluated by comparing age and SE groups. A statistically significant correlation was found between absolute interocular differences in SE and absolute interocular differences in superior mRNFL (*p* = 0.047) and total mRNFL (*p* = 0.040). However, careful examination of the sample included in this study showed that the highest absolute interocular difference in SE to be 3D, followed by 2.125D and 1.75D. In other words, our sample included very few patients with marked differences in interocular SE, thus potentially explaining why we were unable to find a stronger association. Therefore, interocular differences in SE should be explored in daily clinical practice if high levels of asymmetry in GCC values are found. In their study of young Chinese patients assessed using RTVue-100 OCT, Zhou et al. [5] also found that interocular differences in average GCC thickness were significantly correlated with interocular differences in SE in the multiple regression analysis.

The limitations of the present study are mainly related to possible confounding factors influencing the examination (axial length was not measured) [16]. Wang et al. [7] concluded that magnification attributable to axial length and refractive error had minimal impact on measurements of macular and pRNFL. Another limitation may be that all the examinations were performed on the same day and with the same device. However, Cremasco et al. [40] showed that measurements are not significantly affected by these factors.

It would be very interesting to perform further studies including samples of patients with high values of interocular SE asymmetry in order to determine whether SE really affects the remaining GCC measurements. Studies including intraobserver reproducibility could also be performed.

We found that SD-OCT measurements of macular GCC (GCL-IPL and mRFNL) using the Topcon 3D OCT-2000 device (Topcon Corporation, Tokyo, Japan) are reliable and highly reproducible in children aged 5 to 18 years irrespective of age and SE.

## Conclusions

Interocular symmetry is subject to normal variation. Our results contribute to establish reference values for interocular differences in GCL-IPL and mRNFL parameters in children aged 5–18 years using the Topcon OCT device. Interocular asymmetry of SE should be evaluated in order to explain large degrees of asymmetry that can be observed in healthy children.

## Data Availability

The datasets generated and/or analysed during the current study are not publicly available due to the prevision of further publications coming soon, but are available from the corresponding author on reasonable request.
